# C-Reactive Protein Levels at the Midpregnancy Can Predict Gestational Complications

**DOI:** 10.1155/2018/1070151

**Published:** 2018-11-07

**Authors:** Alessandra Vecchié, Aldo Bonaventura, Federico Carbone, Davide Maggi, Antonella Ferraiolo, Beatrice Carloni, Gabriella Andraghetti, Laura Affinito Bonabello, Luca Liberale, Franco Dallegri, Fabrizio Montecucco, Renzo Cordera

**Affiliations:** ^1^First Clinic of Internal Medicine, Department of Internal Medicine, University of Genoa, 6 viale Benedetto XV, 16132 Genoa, Italy; ^2^Diabetology Unit, Department of Internal Medicine, University of Genoa, 6 viale Benedetto XV, 16132 Genoa, Italy; ^3^Department of Obstetrics and Gynecology, IRCCS Ospedale Policlinico San Martino, Genoa, Italy; ^4^Department of Internal Medicine, University of Genoa, 6 viale Benedetto XV, 16132 Genoa, Italy; ^5^Center for Molecular Cardiology, University of Zurich, 12 Wagistrasse, 8952 Schlieren, Switzerland; ^6^IRCCS Ospedale Policlinico San Martino Genoa-Italian Cardiovascular Network, 10 Largo Benzi, 16132, Genoa, Italy; ^7^First Clinic of Internal Medicine, Department of Internal Medicine, University of Genoa, 6 viale Benedetto XV, and Centre of Excellence for Biomedical Research (CEBR), University of Genoa, 9 viale Benedetto XV, 16132 Genoa, Italy

## Abstract

Although essential for a successful pregnancy, a growing body of evidence suggests that maternal inflammation, when dysregulated, may represent a risk factor for both maternal and neonatal outcomes. Here, we assessed the accuracy of maternal C-reactive protein (CRP) concentrations at the middle phase of pregnancy in the identification of maternal adverse outcomes (MAO) until delivery. A correlation between CRP and a complicated pregnancy including both maternal and neonatal adverse outcomes has been investigated, too. In this retrospective study, conducted at the Diabetology Unit of IRCCS Ospedale Policlinico San Martino, Genoa (Italy), 380 outpatient pregnant women have been enrolled at the prenatal visit before performing a 75 g oral glucose tolerance test at 24th-26th gestational week for gestational diabetes mellitus (GDM) screening. Demographic, medical, and reproductive history has been obtained by verbal interview. Data about pregnancy and delivery have been retrieved from medical records. The median value of maternal baseline serum CRP was 3.25 *μ*g/mL. Women experiencing MAO were older, more frequently suffering from hypertension, and showed higher CRP concentrations, with a cutoff value >1.86 *μ*g/mL found by a ROC curve analysis to be accurately predictive for MAO. By a logistic regression analysis, serum CRP levels >1.86 *μ*g/mL have been found to predict MAO also considering maternal age, hypertension, and GDM. Maternal CRP levels have been positively associated with overall pregnancy adverse outcomes (maternal and neonatal), too. In conclusion, in pregnant women serum levels of CRP can early recognize subjects at higher risk for maternal and neonatal complications needing a more stringent follow-up.

## 1. Introduction

Inflammation is an essential element for a successful pregnancy [1]. Indeed, inflammatory processes are involved in implantation and decidualization during early stages of pregnancy, but also in the uterine activation during labor [1–3]. However, in the middle phase of gestation, a quiescence of inflammation is required to ensure maternal tolerance for fetal antigens [4]. A growing body of evidence suggests that a dysregulated maternal inflammation during pregnancy might be a possible risk factor for several neonatal complications [5, 6]. Moreover, some studies have found a correlation between inflammation and the development of gestational complications [7, 8]. The identification of a low-cost and easy-to-measure biomarker of maternal inflammation able to predict risk for complicated pregnancy might allow a better surveillance during gestation.

C-reactive protein (CRP) is routinely used in the diagnosis and clinical monitoring of infections, included those occurring in the obstetric field [9–11]. CRP is also an important biomarker of sterile inflammation, commonly evaluated for monitoring treatment response and predicting long-term outcome in inflammatory diseases [12, 13].

Although the topic has been largely investigated, the purpose of this study is to examine whether maternal serum CRP concentrations in a specific period of the pregnancy—the middle phase—may help in predicting late gestational complications until delivery, considering the large diffusion and the extreme easiness in measuring this biomarker, largely validated in the cardiovascular field.

## 2. Materials and Methods

### 2.1. Study Population and Clinical Assessment

This retrospective study was conducted between October 2012 and November 2014 among 380 consecutive outpatient pregnant women aged 18 or older attending the Diabetology Unit of IRCCS Ospedale Policlinico San Martino (Genoa, Italy) to perform a 75-g oral glucose tolerance test (OGTT) for the screening of gestational diabetes (GDM), as prescribed by current guidelines [14], between the 24th and 26th gestational week (gestational age 24 weeks+0 days – 25 weeks+6 days). The only exclusion criterion was the detection or the clinical suspicion of an active infection. The Ethics Committee of IRCCS Ospedale Policlinico San Martino in Genoa (Italy) approved this protocol, performed in accordance with the guidelines of the Declaration of Helsinki. Patients gave written informed consent before entering the study.

Serum samples were collected during the prenatal visit at baseline before the OGTT and stored in a locked and temperature-controlled freezer at −80°C according to Good Clinical Practice and Good Clinical Laboratory Practice guidelines until analysis [15, 16]. Demographic, medical, and reproductive history was obtained by verbal interview. Data about pregnancy complications, time and type of delivery, and neonatal characteristics at birth were retrieved from medical records.

### 2.2. Definition of Gestational Diabetes Mellitus and Maternal and Neonatal Outcomes

GDM has been defined according to latest American Diabetes Association diagnostic criteria [14]. In the present study, in accordance with a previous study conducted in an Italian cohort [17], maternal adverse outcomes (MAO) included the following conditions: gestational hypertension, premature delivery (before 37th gestational week), and Cesarean delivery. Neonatal outcomes included macrosomia, fetal distress/polyhydramnios, need for neonatal resuscitation, and small and/or large baby size for gestational age (as defined by the upper or lower 10% of Italian birth weight and length percentiles). Neonatal weight and length percentiles were calculated using online neonatal anthropometric charts developed specifically for the Italian population available at http://www.inescharts.com. Ponderal index was calculated by the following equation: (birth weight in grams x 100)/crown-heel length in cubic centimeters [18]; values higher than 2.85 were considered excessive [19].

### 2.3. Study Endpoints

The primary endpoint was to determine whether CRP serum levels could predict the appearance of MAO from the 24th-26th week of gestation until delivery. The secondary endpoint was to evaluate the role of CRP in the association with a complicated pregnancy by considering both maternal and neonatal adverse outcomes.

### 2.4. Detection of Inflammatory, Circulating Biomarker Serum Levels

Serum levels CRP were measured by colorimetric enzyme-linked immunosorbent assay (ELISA) following the manufacturer's instructions (R&D Systems, Minneapolis, MN). The limit of detection was 15.625 pg/mL for CRP. Mean intra- and interassay coefficients of variation were <8%.

### 2.5. Statistical Analysis

Analyses were performed using IBM SPSS Statistics for Windows, Version 23.0 (IBM CO., Armonk, NY). Categorical data are presented as relative and absolute frequencies and compared with Chi-square or Fisher exact test, while continuous variables are shown as median and interquartile range (IQR) and their comparison was done by non-parametric Mann–Whitney *U* test. The prognostic ability of CRP toward the prediction of pregnancy outcomes was evaluated by a receiver operator characteristic (ROC) curve. The area under the curve (AUC) was given with 95% confidence interval (CI) and the cutoff point of CRP was calculated maximizing the sensitivity in accordance with the Youden's index. The predictive ability of CRP toward MAO was calculated by a logistic regression. The association between CRP and overall pregnancy adverse outcomes (both maternal and neonatal) was calculated by a linear regression. In the multivariate model, we adjusted for maternal age at the time of OGTT, presence of hypertension, and the development of GDM. For all statistical analyses, a 2-sided *p*-value <0.05 was considered as statistically significant.

## 3. Results

### 3.1. Patients' Characteristics

Baseline characteristics of the overall cohort are shown in Table 1. Pregnant women median age at the time of enrollment was 34 (31-37), with a remarkable prevalence of Caucasian patients (90.7%). Median body mass index (BMI) before pregnancy was 21.19 (19.82-26.63), with a median weight gain at delivery of 12 kg (10-15). In 45 women (11.9%), glucose values at different time points of OGTT met criteria for GDM. Most frequent complications among women were Cesarean delivery (50.6%) and premature delivery (9.4%). Among babies, most frequent adverse outcomes included small size for gestational age (22.4%), macrosomia (2.9%), and the need for resuscitation (1.7%), as shown in Table 1. The median value of maternal serum CRP was 3.25 *μ*g/mL (1.61-8.07).

### 3.2. CRP Serum Levels and Maternal Adverse Outcomes

We then evaluated the characteristics of the overall cohort by comparing patients according to the presence or absence of MAO (Table 2). Women who experienced MAO were older and more frequently suffered from hypertension. When analyzing inflammatory mediators, CRP serum levels were significantly higher in the MAO group and babies born from mothers with MAO were smaller (Table 2). Apgar scores of babies born from mothers with MAO were lower; accordingly, all cases of neonatal resuscitation occurred in this group (Table 2).

### 3.3. High CRP Serum Levels and Prediction of Maternal and Neonatal Adverse Outcomes

**Figure 1:**
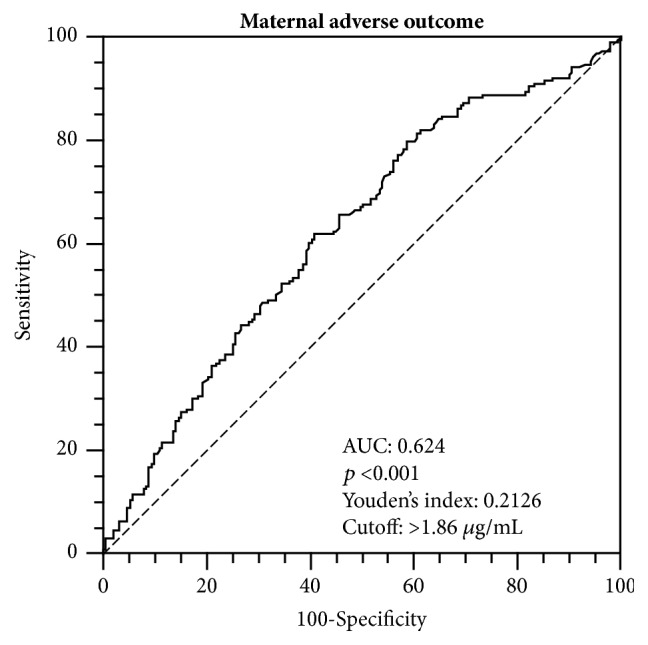
The receiver operator characteristic (ROC) curve analysis for C-reactive protein towards the occurrence of maternal adverse outcomes from the second trimester of pregnancy until delivery is shown.

By a ROC curve analysis, we found maternal CRP serum levels during pregnancy to hold significant prognostic accuracy towards MAO occurrence (AUC 0.624 [95% CI 0.573-0.672]; *p*< 0.001, Figure 1). A CRP value >1.86 *μ*g/mL was found as the best cutoff point, having a sensitivity of 79.9% and a specificity of 41.4% (Figure 1).

By a logistic regression analysis, we could show serum CRP levels >1.86 *μ*g/mL to predict MAO occurrence (OR 2.80, 95% CI 1.77-4.43, *p*<0.001), as shown in Table 3. This result was confirmed also in the multivariate model considering maternal age, hypertension, and the development of GDM during pregnancy (OR 3.73, 95% CI 2.02-6.86, *p*<0.001, and Table 3). We further investigated the impact of CRP on all adverse outcomes, both maternal and neonatal. Indeed, CRP values were positively associated with pregnancy adverse outcomes both in the univariate (*β*=0.043, 95% CI 0.003-0.083,* p=*0.036) and in the multivariate model when maternal age, hypertension, and development of GDM were considered (*β*=0.05, 95% CI 0.010-0.090, *p*=0.015, Table 4).

## 4. Discussion

The main finding of this study is that maternal serum levels of CRP during the second trimester of pregnancy represent a useful predictor of MAO occurrence. Moreover, a positive association between maternal CRP levels and the composite endpoint including both maternal and neonatal adverse outcomes has been demonstrated. These results are in accordance with prior studies exploring the association between CRP and pregnancy adverse outcomes [20–25]. In particular, Sorokin and colleagues found an association between maternal CRP (median level 68.52 *μ*g/mL) and preterm delivery in 495 pregnant women at increased risk for spontaneous preterm delivery who were already administered with corticosteroids [22]. Similarly, Pitiphat et al. demonstrated that women with CRP serum levels ≥8 mg/L had a greater risk of preterm delivery [21]. Differently from other studies [20–23], we found a lower CRP threshold above which adverse events, especially the maternal ones, complicated the pregnancy. Ertas and coworkers described a CRP cutoff of 9.66 mg/L, which was associated with the severity of the clinical risk of pre-eclampsia and with adverse neonatal outcome [20]. Since these levels are extremely higher than ours, a potential explanation might be the presence in our cohort of cases of gestational hypertension only, while no woman developed pre-eclampsia, thus limiting the comparison of results. Moreover, CRP has been measured at different time points. While Pitiphat et al. and Tjoa et al. measured CRP during the first trimester, Ertas et al. and Sorokin et al. evaluated it between second and third trimester. Indeed, inflammation is known to increase early for implantation and then to reduce during the middle phase of pregnancy due to maternal tolerance to fetal antigens [26]. Taken together, when compared to previous investigations, our findings are likely to highlight that even a little increase in the degree of systemic inflammation can represent an additional risk for the development of pregnancy complications, both for mother and baby.

Additionally, in a recent study women who had a preterm delivery or gave birth to a baby small for gestational age showed an increased risk for premature cardiac disease or death [27]. This finding is of great interest, suggesting a possible correlation between the inflammatory* milieu* of the middle phase of gestation, associated with adverse pregnancy outcomes, and the inflammatory burden of women's rest of life, usually correlated with the development of cardiovascular diseases. This evidence has a practical consequence because the identification of women with a higher degree of inflammation during gestation could help in preventing not also pregnancy complications, but also future cardiovascular events.

Age is a well-known risk factor for the development of MAOs during pregnancy [28–30]. Similarly, GDM and gestational hypertension are associated with an increased risk of maternal and neonatal adverse outcomes, especially in case of inadequate glycemic and pressure control [30, 31]. Especially GDM is a recognized risk factor for pregnancy-related complications [32] affecting both the mother by increasing the incidence of pre-eclampsia, gestational hypertension, and polyhydramnios [33] and the fetus by favoring the occurrence of macrosomia, prematurity, need for neonatal intensive care unit admission, and congenital malformations [34, 35]. In our cohort, a positive association between serum CRP levels and the development of maternal complications persisted even when maternal age, GDM, and gestational hypertension have been taken into consideration. An explanation may be found in the limited number of women suffering from both GDM and gestational hypertension. These findings encourage the routine CRP dosage to better stratify pregnant women at high risk of complications, independently of the presence of other known risk factors.

Some limitations have to be acknowledged in our paper. First of all, data on lifestyle habits and cardiovascular risk factors, such as cholesterol levels and smoking habits, were not available for the analysis and may have partially influenced adverse outcomes. Secondly, serum samples have been collected only at the time of OGTT and we could not evaluate possible changes of CRP until delivery. Finally, data have been retrieved in a single center and this may limit the generalization of our results, needing future multicenter studies to confirm them on a larger number of patients. Finally, although the clinical occurrence of infections has been excluded, we cannot ensure the absence of asymptomatic infections potentially altering CRP levels and influencing pregnancy as no urine or blood culture has been performed.

## 5. Conclusions

Maternal CRP serum levels might be an effective and convenient tool to recognize women at high risk for pregnancy complications, thus needing a closer follow-up. Further studies are warranted in order to widen our knowledge about pathophysiological mechanisms linking inflammation and pregnancy complications, in particular by considering the real impact of GDM in determining both maternal and neonatal outcomes. In this view, we are planning to consider only women with GDM in order to establish clearer correlations between diabetes and adverse outcomes, maybe evaluating glycemic values since the gestation beginning.

## Figures and Tables

**Table 1:** Baseline characteristics of the overall cohort.

	**Overall cohort (n=380)**
**Demographic and clinical characteristics**	

Age at enrollment, years (IQR)	34 (31-37)
CRP^*∗*^, *μ*g/mL (IQR)	3.25 (1.61-8.07)
Ethnicity	
Caucasian, n (%)	342 (90.7)
Latin American, n (%)	20 (5.3)
African, n (%)	6 (1.6)
Indian, n (%)	9 (2.4)
BMI^§^, kg/m^2^ (IQR)	21.19 (19.82-26.63)
Underweight, n (%)	22 (9.2)
Normal weight, n (%)	176 (73.6)
Overweight, n (%)	37 (15.5)
Obese, n (%)	10 (4.1)
Weight gain at delivery, kg (IQR)	12 (10-15)

**Comorbidities**	

Thyroid disease, n (%)	28 (7.4)
Hypertension, n (%)	8 (2.1)
PCOS^¥^, n (%)	7 (1.8)
Coagulation/PLT^Φ^ diseases, n (%)	8 (2.1)
Autoimmune diseases, n (%)	35 (9.2)
GDM^#^, n (%)	45 (11.9)

**Therapy**	

Thyroid hormone replacement therapy, n (%)	27 (7.1)
Corticosteroid hormone, n (%)	9 (2.4)
Aspirin, n (%)	16 (4.2)

**Pregnancy and delivery characteristics**	

Gestational hypertension, n (%)	5 (1.3)
Premature delivery, n (%)	32 (9.4)
Cesarean delivery, n (%)	177 (50.6)
Urgent Cesarean delivery, n (%)	40 (11.4)
Gestational age at delivery, days (IQR)	275 (270-282)
Overall maternal adverse outcomes	189 (49.7)

**Baby characteristics**	

Baby weight, g (IQR)	3280 (2948.75-3571.25)
Baby length, cm (IQR)	49 (48-50)
Small for gestational age, n (%)	74 (22.4)
Large for gestational age, n (%)	23 (6.9)
Ponderal index (IQR)	2.75 (2.56-2.94)
Macrosomy, n (%)	10 (2.9)
Apgar score	
0, n (%)	1 (0.3)
3, n (%)	1 (0.3)
4, n (%)	1 (0.3)
5, n (%)	1 (0.3)
6, n (%)	1 (0.3)
7, n (%)	2 (0.6)
8, n (%)	4 (1.2)
9, n (%)	23 (6.8)
10, n (%)	306 (90)
Resuscitation, n (%)	6 (1.7)
Fetal distress/polyhydramnios, n (%)	3 (0.8)
Overall neonatal adverse outcomes, n (%)	173 (45.5)
Maternal and neonatal adverse outcomes, n (%)	272 (71.6)

Data are expressed as median (interquartile range [IQR], number [n], or percentage [%]).

*∗* CRP: C-reactive protein.

§ BMI: body mass index.

¥ PCOS: polycystic ovary syndrome.

Φ PLT: platelets.

# GDM: gestational diabetes mellitus.

**Table 2:** Characteristics of the cohort according to maternal adverse outcomes.

	**No maternal adverse outcomes (n=191)**	**Maternal adverse outcomes (n=189)**	***p***
**Demographic and clinical characteristics**			

Age at enrollment, years (IQR)	33 (30-36)	35 (32-38)	**<0.001**
CRP^*∗*^, *μ*g/mL (IQR)	2.51 (1.16-6.11)	4.40 (2.03-10.1)	**<0.001**
Ethnicity			0.102
Caucasian, n (%)	167 (88.4)	175 (93.1)	
Latin American, n (%)	11(5.8)	9 (4.8)	
African, n (%)	4 (2.1)	2 (1.1)	
Indian, n (%)	7 (3.7)	2 (1.1)	
BMI^§^, kg/m^2^ (IQR)	21.01 (19.79-22.95)	21.48 (19.83-24.22)	0.145
Underweight, n (%)	12 (9.7)	10 (8.7)	
Normal weight, n (%)	96 (77.4)	80 (69.6)	
Overweight, n (%)	12 (9.7)	19 (16.5)	
Obese, n (%)	4 (3.2)	6 (5.2)	
Weight gain at delivery, kg (IQR)	13 (11-16)	12 (9-15)	0.179

**Comorbidities**			

Thyroid disease, n (%)	12 (6.3)	16 (8.5)	0.416
Hypertension, n (%)	1 (0.5)	7 (3.7)	**0.031**
PCOS^¥^, n (%)	1 (0.5)	6 (3.2)	0.055
Coagulation/PLT^Φ^ diseases, n (%)	2(1)	6 (3.2)	0.149
Autoimmune diseases, n (%)	17 (8.9)	18 (9.5)	0.834
GDM^#^, n (%)	18 (9.5)	27 (14.3)	0.153

**Therapy**			

Thyroid hormone replacement therapy, n (%)	12 (6.3)	15 (7.9)	0.531
Corticosteroid hormone, n (%)	4 (2.1)	5 (2.6)	0.724
Aspirin, n (%)	4 (2.1)	12 (6.3)	**0.039**

**Pregnancy and delivery characteristics**			

Gestational hypertension, n (%)	0	5 (2.6)	**0.024**
Premature delivery, n (%)	0	32 (17.4)	**<0.001**
Cesarian delivery, n (%)	0	177 (93.7)	**<0.001**
Urgent caesarian delivery, n (%)	0	40 (21.2)	**<0.001**
Gestational age at delivery, days (IQR)	280 (273-285)	273 (267-279)	**<0.001**

**Baby characteristics**			

Baby weight, g (IQR)	3300 (3040-3595)	3210 (2860-3535)	**0.046**
Baby length, cm (IQR)	49 (48-51)	49 (47-50)	**0.015**
Small for gestational age, n (%)	38 (24.5)	36 (20.6)	0.392
Large for gestational age, n (%)	7 (4.5)	16 (9.1)	0.097
Ponderal index (IQR)	2.76 (2.55-2.97)	2.75 (2.56-2.94)	0.898
Macrosomy, n (%)	4 (2.4)	6 (3.2)	0.660
Apgar score			**<0.001**
0, n (%)		1 (0.6)	
3, n (%)		1 (0.6)	
4, n (%)		1 (0.6)	
5, n (%)		1 (0.6)	
6, n (%)		1 (0.6)	
7, n (%)		2 (1.1)	
8, n (%)		4 (2.2)	
9, n (%)	5 (3.1)	18 (10)	
10, n (%)	155 (96.9)	151 (83.9)	
Resuscitation, n (%)	0	6 (3.2)	**0.022**
Fetal distress/polyhydramnios, n (%)	1 (0.5)	2 (1.1)	0.557
Overall neonatal adverse outcomes, n (%)	83 (43.5)	90 (47.6)	0.416

Data are expressed as median (interquartile range [IQR], number [n], or percentage [%]).

*p*-values were calculated according to Chi-square or Fisher exact test or Mann-Whitney *U* test when appropriate and referred to as comparison between study groups.

Statistically significant correlations are highlighted in bold character.

*∗* CRP: C-reactive protein.

§ BMI: body mass index.

¥ PCOS: polycystic ovary syndrome.

Φ PLT: platelets.

# GDM: gestational diabetes mellitus.

**Table 3:** Logistic regression model showing the predictive value of C-reactive protein cutoff (>1.86 *μ*g/mL) toward overall maternal outcomes.

	Univariate model			Multivariate model		
	OR^*∗*^	95% CI^§^	*p*-value	OR	95% CI	*p*-value
**Maternal adverse outcome**						

CRP^*¥*^ >1.86 *μ*g/mL	2.80	1.77-4.43	**<0.001**	2.93	1.83-4.69	**<0.001**
Age				1.09	1.04-1.14	**0.001**
GDM^Φ^				1.37	0.70-2.65	0.358
Hypertension				5.15	0.58-46.04	0.142

Statistically significant correlations have been highlighted in bold character.

*∗* OR: odds ratio.

§ CI: confidence interval.

¥ CRP: C-reactive protein.

Φ GDM: gestational diabetes mellitus.

**Table 4:** Linear regression showing the association between C-reactive protein and overall adverse outcomes (both maternal and neonatal).

	Univariate model			Multivariate model		
	*β*	95% CI^*∗*^	*p*-value	*β*	95% CI	*p*-value
**Overall adverse outcome**						

CRP^§^	0.043	0.003-0.083	**0.036**	0.050	0.010-0.090	**0.015**
Age				0.007	-0.003-0.016	0.168
GDM^¥^				0.129	-0.010-0.268	0.068
Hypertension				0.250	-0.066-0.567	0.121

Statistically significant *p* values are displayed in bold characters.

*∗* CI: confidence interval.

§ CRP: C-reactive protein.

¥ GDM: gestational diabetes mellitus.

## Data Availability

Due to ethical committee permission we have to protect also anonymized data and ask for an Ethical Committee permission if database is required for checking.
